# Relationship between agro-environmental variables and breeding Hylids in rice paddies

**DOI:** 10.1038/s41598-018-26222-w

**Published:** 2018-05-23

**Authors:** Amaël Borzée, Kyongman Heo, Yikweon Jang

**Affiliations:** 1 Laboratory of Behavioral Ecology and Evolution, School of Biological Sciences, Seoul National University, Seoul, 08826 Republic of Korea; 2 College of Natural Science, Sangmyung University, Seoul, 03016 Korea; 3 Department of Life Sciences and Division of EcoScience, Ewha Womans University, Seoul, 03760 Republic of Korea; 4 Interdisciplinary Program of EcoCreative, Ewha Womans University, Seoul, 03760 Republic of Korea

## Abstract

When natural wetlands are destroyed, many anuran species are forced to breed in alternative habitats such as rice paddies. We conducted field surveys for the endangered *Dryophytes suweonensis* and the numerous *D. japonicus*, from the beginning of the breeding season until two weeks after its peak. We recorded the presence, number of individuals and calling indices for each species. We hypothesized that *D. japonicus* would start breeding earlier than *D. suweonensis*, which would have originally been breeding solely in floodplains. The results of our analyses demonstrate that the rice cultivation phase was the most important factor in predicting the calling activities of both species. Furthermore, peak calling activities of both species matched the optimal hydroperiod in rice paddies. In addition, *D. japonicus* breeding behaviour was influenced by environmental variables such as temperature, whereas *D. suweonensis* seemed to require the planting of rice seedlings to initiate breeding. Therefore, as both *Dryophytes* species’ breeding activities are influenced by agro-environmental variables, this study highlights the importance of also preserving anthropogenically modified landscapes for the conservation of species.

## Introduction

Most amphibian species are found in wetlands, typically situated in low-lying plains^1–3^. Unfortunately, low-lying plains, along with their wetlands, are the optimal environment for large-scale rice cultivation. Worldwide, the primary driver of wetland loss is the conversion of land to agricultural fields^4,5^. For instance, 50% of wetlands were converted to agricultural fields in North America and Europe^6^, and 90% of wetlands were converted in Brazil^7^. In 2003, Asia accounted for approximately 89% of all rice paddies in the world^8^. Of the 22% of landmass available for crop production in the Republic of Korea, 90% of arable lands are used for rice production^9^. Wetlands that are not used for agriculture are often targeted for urbanisation, as alluvial plains are attractive to both humans and other species^10,11^.

Amphibians are opportunistic breeders^12–16^, and extensive agricultural land expansion has caused wetland breeders to become rice paddy breeders. Rice paddies present some advantages for breeding amphibians, including a prolonged annual hydroperiod. In fact, many amphibian species are solely or heavily reliant on rice paddies for breeding^7,17–21^. Nevertheless, the conservation of natural wetlands is a necessity as some species are restricted to a wetland habitat^22,23^, or do not fare as well in rice paddies^24,25^. The decrease in natural wetlands highlights the need for surrogate habitats for wetland-based amphibians^26,27^. It is also worth considering that, with planning, rice paddies can be used as a connecting matrix between populations breeding at natural sites^7^, and therefore limit the effects of fragmentation arising from human development^28–30^.

Some agricultural areas used for rice cultivation boast a relatively high diversity of species^4,21,31–35^, such as wading birds in the Mediterranean basin^36^, and amphibians in Brazil^7^. However, variations in rice paddy management can have a different impact on amphibian populations across regions. For instance, flooding of rice paddies during the fallow phase did not alter amphibian richness in Brazil^7^, but it did in California^32,37^. Asia, with the largest rice plantations in the world, sees the same association between rice cultivation and anuran biomass^16,20,38^, although the amphibian biomass and species richness in the same areas before agricultural development is largely unknown. However, for documented cases such as *Rana japonica* and *R. porosa* in Japan, the population sizes of these species are negatively affected by modern farming practices, in comparison to traditional farming^18^.

Anuran assemblages are strongly influenced by abiotic and biotic factors^39–43^. Here, we try to understand whether and how the breeding biology of Korean treefrogs is limited by farming practices including water availability at rice paddies. Land use conversion to rice paddies since the development of human agriculture (such as described by Fuller *et al*.^44,45^) might have affected the functional equilibrium between the endangered *Dryophytes suweonensis* and the numerous *D. japonicus*^46^, a genus previously attributed to *Hyla*^47^. It is highly likely that *D. japonicus* relies on forested habitats during the non-breeding season^48^, while *D. suweonensis* might be restricted to wetlands for both breeding and non-breeding seasons^49^. Therefore, prior to rice farming, lowlands would have provided most of the habitats for *D. suweonensis*, which requires shallow and lentic water bodies^50^.

The new alternative breeding ground provided by rice paddies was readily colonized by *D. japonicus* due to its wide acceptance of habitats^20,51^, resulting in increases in population sizes^46^. Thus, the two treefrog species were forced to breed together in rice paddies, despite their segregated evolutionary history, evident by their different responses to predators^52^. We hypothesized that *D. japonicus* would start breeding earlier than *D. suweonensis*, as it originates from habitats with a reduced hydroperiod and is evolutionarily advantaged by timely exploiting all water resources available. Thus, the secondary purpose of this study was to assess the viability of rice paddies and to understand the impact of agricultural practices on the survival of the endangered *D. suweonensis* and the numerous *D. japonicus*.

## Material and Methods

This study began prior to the breeding seasons of both *Dryophytes japonicus* and *D. suweonensis*^20,53^. The setting of modern rice paddies leads to a specific grouping of rice paddies, here referred to as the rice-paddy complex, with a central lane that follows the central ditch running mostly straight through the complex for irrigation purposes. This lane is usually along the longest and straightest line available, and follows the centre of the valley used for the rice-paddy complex. Rice paddies are delimited by levees approximately 40 cm wide and 20 to 60 cm high, covered with grasses used by amphibians for basking, foraging, and sheltering^18,54^. In the Republic of Korea, *Dryophytes spp*. and *Pelophylax spp*. are typically found together on levees during their breeding seasons^54^.

**Figure 1:**
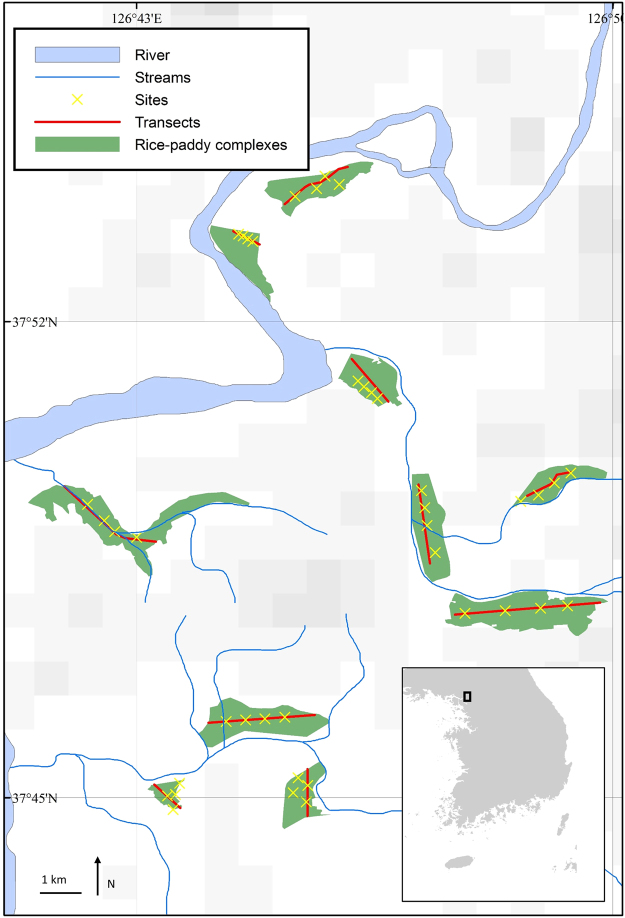
Representative map of the 40 sites and 10 complexes where data collection took place over a 60-day study period, starting on 16 April 2014. All sites are within the administrative area of Paju, Gyeonggi Province, Republic of Korea. The map was generated in ArcMap 10.5 (Esri, Redlands, USA; https://www.esri.com).

Ten rice-paddy complexes where *D. suweonensis* was known to occur were selected as study sites^53,55,56^. However, *D. suweonensis* was not heard calling at two of the selected complexes (Fig. 1). Because the advertisement calls of *D. japonicus* and *D. suweonensis* are species-specific^57^, we employed acoustic monitoring to assess the population sizes for the two species^58–62^.

Surveys were conducted twice a week over a 60-day study period, starting on 16 April 2014 in the city of Paju, Gyeonggi Province, Republic of Korea (Fig. 1), and resulted in 15 surveys per site during the study period. A site was defined as a locality where surveys were conducted for four survey points, in addition to which there was a walking survey at the scale of the rice-paddy complex. Two sampling techniques were used for acoustic monitoring: transect and point surveys. Each of the 10 sites were surveyed using one transect and one independent point survey at each of four individual rice paddies. Each point survey was at least 500 m away from any other point survey to insure independence of data, as an individual *D. suweonensis* can be heard up to 250 ± 45 m away^56^.

For both point and transect surveys, we documented the phases of rice cultivation: fallow, ploughed, agricultural flood, tilled, and rice plantation. Two other variables were annotated separately, due to their non-cyclical and random occurrence: pre-seedling production and rainwater partial flooding. Pre-seedling production refers to the period when all seedlings are grown together in restricted areas, under tarpaulins for protection against the cold, and usually in a single low lying rice paddy for ease of irrigation. These agricultural processes were deemed important for the breeding of the two *Dryophytes spp*. due to the accompanying disturbance by agricultural equipment such as tractors, the hydroperiod^42^, and diversity and abundance of juvenile amphibians^39^. Finally, plantation of rice seedlings may be beneficial for male *D. suweonensis*, as they use them as perches from which to call^63^. At the beginning of each survey, we also recorded date, time of day, air temperature (°C) and relative humidity (%) due to their importance for call production^40^. Data for two rice-paddy complexes could not be recorded for the ninth survey, and are treated as missing data for subsequent analyses.

### Point surveys

Aural point surveys were conducted at each of the 40 individual rice paddies. After arrival at a survey point, 5 min were spent waiting quietly and the next 5 min were used to record the calling activity of hylids. All calling individuals heard were recorded, although some were potentially outside of the focal rice paddy. In previous studies, 5 min has been shown to be sufficient to monitor the calling activities of many species^59,61,64^. The calling activity was recorded in two ways: first we counted the number of calling individuals within the rice paddy selected. This procedure was repeated a minimum of 12 times to ensure that all calling individuals were accounted for. This method was deemed adequate for *D. suweonensis* as the species is not abundant, *i.e*. the maximum number of individuals per survey point was 10 (mean = 0.21, SD = 0.72), and advertisement calls are clear and unique. For *D. japonicus*, however, calling males regularly exceeded 10 individuals, and this method was not adequate. Consequently, the maximum number of individuals counted was set at 10 for further analyses, based on the maximum number of *D. suweonensis* individuals recorded. Instead, we relied on the calling index (CI) as defined by Mossman, *et al*.^65^ and modified by Roh, *et al*.^53^. The number of calling individuals is classified in four categories: 0: no individuals are calling; 1: calls are not overlapping and it is possible to count the number of individuals calling; 2: calls are overlapping but it is still possible to count the number of individuals calling; 3: it becomes impossible to count the number of individuals. This method was appropriate for the numerous *D. japonicus*, but not optimal for the low calling numbers of *D. suweonensis*. Consequently, the results of both methods were analysed jointly and separately.

### Transect surveys

Following the point survey of all four points at a rice-paddy complex, we moved to one end of the central lane of the same rice-paddy complex. The line transect surveys were conducted at a maximum speed of 80 m/min for a minimum of 10 min (~5 km/h, brisk walking) along the central lane. The presence or absence of individuals was binary encoded for each species. The line transects were not centrally located in complex 6 and 10 (Fig. 1), but they were still within hearing ranges of the entire rice-paddy complexes. We empirically tested for aural detection of the two species (*n* = 20) by measuring the distance at which calling males could be heard calling, in different weather conditions. The detectability of these two species was determined to at 250 m (±45), and thus adequate for the protocol followed.

### Statistical analysis

The first set of analyses determined the relationship between agro-environmental variables and the calling activity of the two species at two scales: survey point and rice-paddy complex. To do so, we numerically encoded “cultivation phase” into an ordinal variable ranging from 1 to 5, matching with fallow phase, ploughed, agricultural flood, tilled and rice plantation, respectively. Additionally, we binary encoded pre-seedling production and rainwater partial flooding. To test whether environmental and agricultural variables were important for the production of advertisement calls by both species, we tested for species occurrence, calling activity and CI. Regression or General Linear Models (GLM) with the same dependent variables were used, depending on the response variables.

#### Binomial regression

The occurrence of *D. japonicus* and *D. suweonensis* was analysed using binary logistic regressions at two spatial scales: survey points and line transects. The occurrence of the two species was binary encoded at both scales. Thus, there were four separate binary logistic regressions. Additionally, all independent variables were on either continuous, ordinal or nominal scales: temperature, humidity, cultivation phase, pre-seedlings, rain flood and survey points. All variables measured were spatially or geographically independent of each other. We did not include season and time of day in this analyses as the surveys had been designed to increase detection based on temporal and seasonal detectability.

#### Generalised Linear Model

When determining the statistical test for the calling activities of *D. suweonensis*, assessed here as the number of calling individuals, we determined normality with the Kolmogorov-Smirnov test with Lilliefors Significance Correction (*D*_592_ = 491, *p* < 0.001), the distribution of residuals with QQ plots, and determined the homogeneity of variance with Levene’s test (*F*_1,590_ = 149.40, *p* = 0.059). We observed four outliers through the analysis of box-plots for the number of calling *D. suweonensis*. We chose to ignore this violation of assumptions for Linear Models as it only included four out of 592 data points, and selected a Generalised Linear Model with an ordinal logistic response variable for the number of calling *D. suweonensis*. The Generalised Linear Model was run with factor and covariates set under a main effect model. The predictor variables were temperature, humidity, and cultivation phase as covariates, and pre-seedlings, rain flood and survey points nested within rice-paddy complexes, and rice-paddy complexes as factors. All variables were spatially or geographically independent of each other, and we did not include season and time of day in these analyses as the surveys had been designed to increase detection based on temporal and seasonal detectability.

#### GLM

The calling activity of *D. japonicus*, inferred through the Calling Index was analysed through a General Linear Model (GLM). Independent categorical variables (pre-seedlings, rain flood and survey points nested within rice-paddy complexes and rice-paddy complexes) were set as fixed factors and independent linear variables (temperature, humidity, and cultivation phase) as covariates. When testing for assumptions for the model, we did not notice any outliers through the analysis of box-plots. In addition, we determined the normal distribution of the data through the graphical analysis of residuals on QQ plots, and determined the homogeneity of variance with Levene’s test for homogeneity of variances (*F*_(1,590)_ = 28.77, *p* = 0.051).

#### Repeated measure ANOVA

We also analysed the dataset with the purpose of segregating the calling activity in pre-, post-, and lekking periods for further analysis, as the effect of environmental variables changes based on lekking periods^66–68^. We employed a repeated measure ANOVA with survey as the predictor variable to assess the patterns of temporal variations in calling activity of the two species. Because the number of calling *D. suweonensis* was significantly correlated with all individual counts and presence variables (*P* < 0.001, *n* = 588; *R* > 0.18), the number of calling *D. suweonensis* was used as the response variable representative of the lekking activity for both species. We tested for homogeneity of variance with Levene’s test and sphericity assumption with Mauchy’s test. We ran the repeated measures ANOVA with the Greenhouse-Geisser correction^69^ due to the violation of the assumption of sphericity. Furthermore, compound symmetry (homogeneity of the variance-covariance matrix) was assumed for this analysis. The analysis was set with 15 levels of within subject variables and the comparison of main effects. The repeated measure ANOVA for the number of calling *D. suweonensis* for each survey demonstrated that the mean calling activity differed significantly between surveys (*F* = 6.06; *df* = 3.46; P < 0.001). *Post-hoc* analyses on a case by case basis, through the comparison of main effects (Supplementary Materials 1), showed that surveys 8 to 13 were grouped together in a non-significantly different group (mean *p* = 0.28), although significantly different from other surveys during the same period (mean *p* = 0.05). This highlights the peak activity of the breeding season, and there was no significant variation in the number of individuals detected through aural surveys for *D. suweonensis*. We therefore qualified replicates 1 to 7 as *pre-lekking*, replicates 8 to 13 as the *lekking period* and replicates 14 and 15 as *post-lekking*, following Kim^68^.

Following the division of the lekking period, pre- and post-lekking were analysed through binary logistic regressions for each species using the presence data at the complex as dependent variables. This choice of dependent variable was made due the fact that the CI is primarily 0 and 1 at this time period, the number of individuals for a survey point is too conservative for *D. japonicus* and presence in the complex described more variation than at a single survey point. The variables used for the pre-lekking analysis were season, temperature, humidity and cultivation phase. For the post-lekking analysis, only season, temperature, humidity and agricultural flooding were used as all other variables were constant, *e.g*. agricultural practices such as tilling are required only once per season.

The lekking period was analysed through multinomial logistic regressions, based on the CI of the species. CI was set as the dependent variable as it was assessed to be the best fitting factor due to the high variability for this time period, *i.e*. CI from 0 to 3. The cultivation phase was set as factors, while season, temperature and humidity were set as covariates, under a main effect model.

In addition, we plotted all significant variables from any of the analyses against the calling activity of the two species to obtain a graphical representation of the variables important for the calling activity of the two species, for the totality of the breeding season. We used the presence/absence of the species at survey points, and not the number or CI, as a single individual calling was assessed to be representative of the breeding status of the species due to the commonalities of the physiological processes involved^70^, and the absence of outliers that would have falsified our analysis. This differs in the previous analysis based on the CI, which was limited to the lekking period only.

Finally, we assessed the relationship between the two species to ensure that their calling activities were not influencing each other, and that agricultural practices were the reasons for the patterns described. This was examined because *D. japonicus* is known to significantly influence *D. suweonensis* calling site selection^63^. The dataset was not collected in a way that enables statistical tests to fully address the question, but correlations were tested for consistency. We used Pearson correlations to compare the calling activity of the two species, independently for each of the 15 replicates. All analyses were conducted in SPSS (IBM SPSS Statistics Inc., Chicago USA).

## Results

Environmental conditions changed drastically as the rice cultivation phases progressed. During the study period, the average temperature when no treefrogs were surveyed at the rice-paddy complex scale was 13.88 ± 5.58 °C (mean ± SD) for *D. suweonensis* and 13.70 ± 6.56 °C for *D. japonicus*. The temperature was on average higher for *D. suweonensis* (19.78 ± 4.00 °C) than for *D. japonicus* (16.91 ± 5.16 °C) when the two species were present. The pattern was the same for temperature and occurrence at single survey points for both species, and also for humidity at the two scales and for both species. However, temperature and humidity were similar during fallow phase (11.62 ± 2.33 °C; 73.62 ± 9.54% rH), ploughed phase (11.50 ± 3.34 °C; 61.51 ± 13.41% rH) and tilling (11.70 ± 2.74 °C; 61.53 ± 13.54% rH), while temperatures were generally higher during flattening (18.20 ± 5.88 °C) and agricultural flooding (20.53 ± 3.97 °C). In comparison, the relative humidity was not generally different between flattening (75.43 ± 17.43% rH) and agricultural flooding (84.61 ± 11.03% rH).

### Environmental and agricultural variations

#### Binomial regressions

**Figure 2:**
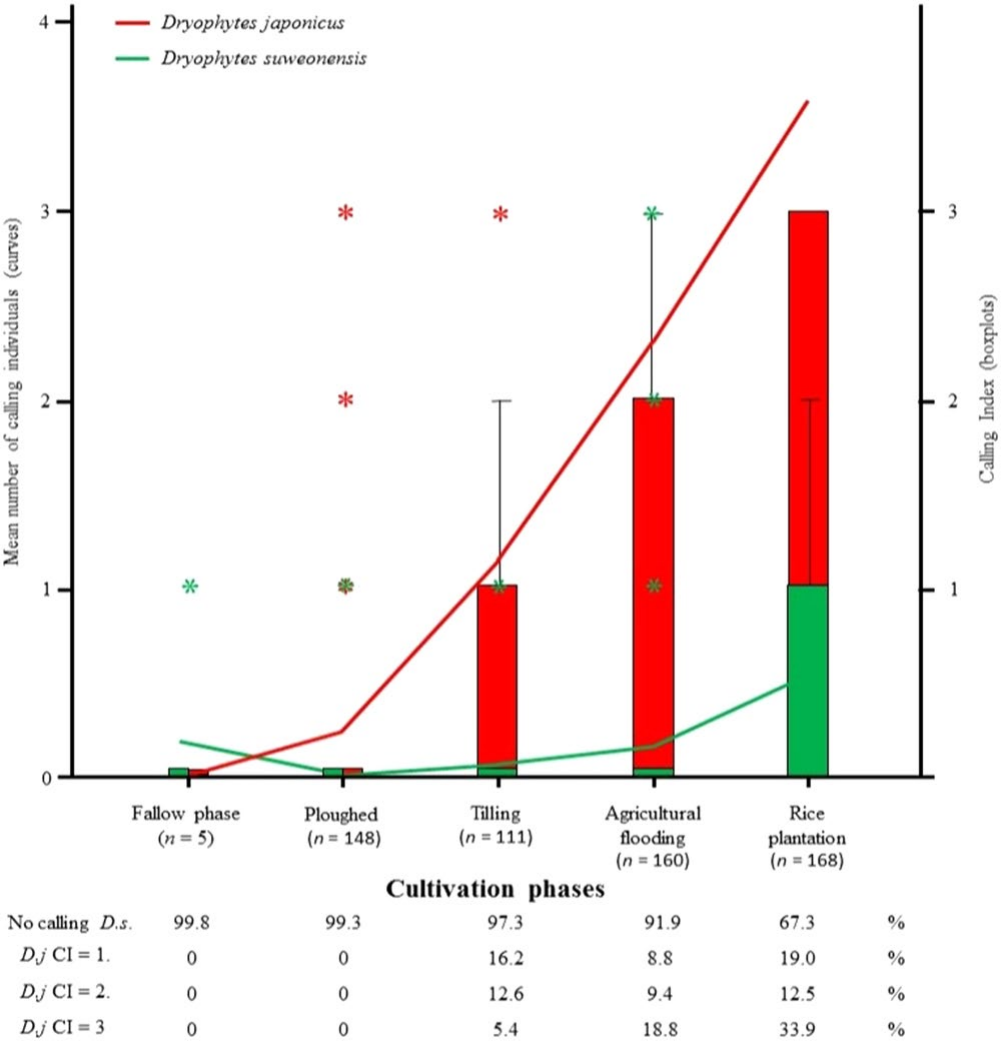
Relationship between cultivation phase and the number of calling individuals for *Dryophytes suweonensis* (=*D.s*) and CI for *D. japonicus* (=*D.j*). The calling index and sites without calling individuals are in %. Stars denote outliers. The 50% median for *D. japonicus* is CI = 0 for tilling and agricultural flooding, and CI = 1 for rice plantation. For *D. suweonensis*, the 50% median is CI = 0 for rice plantation. No whiskers are visible when values reach extreme CIs (*i.e*. 1 or 3).

**Figure 3:**
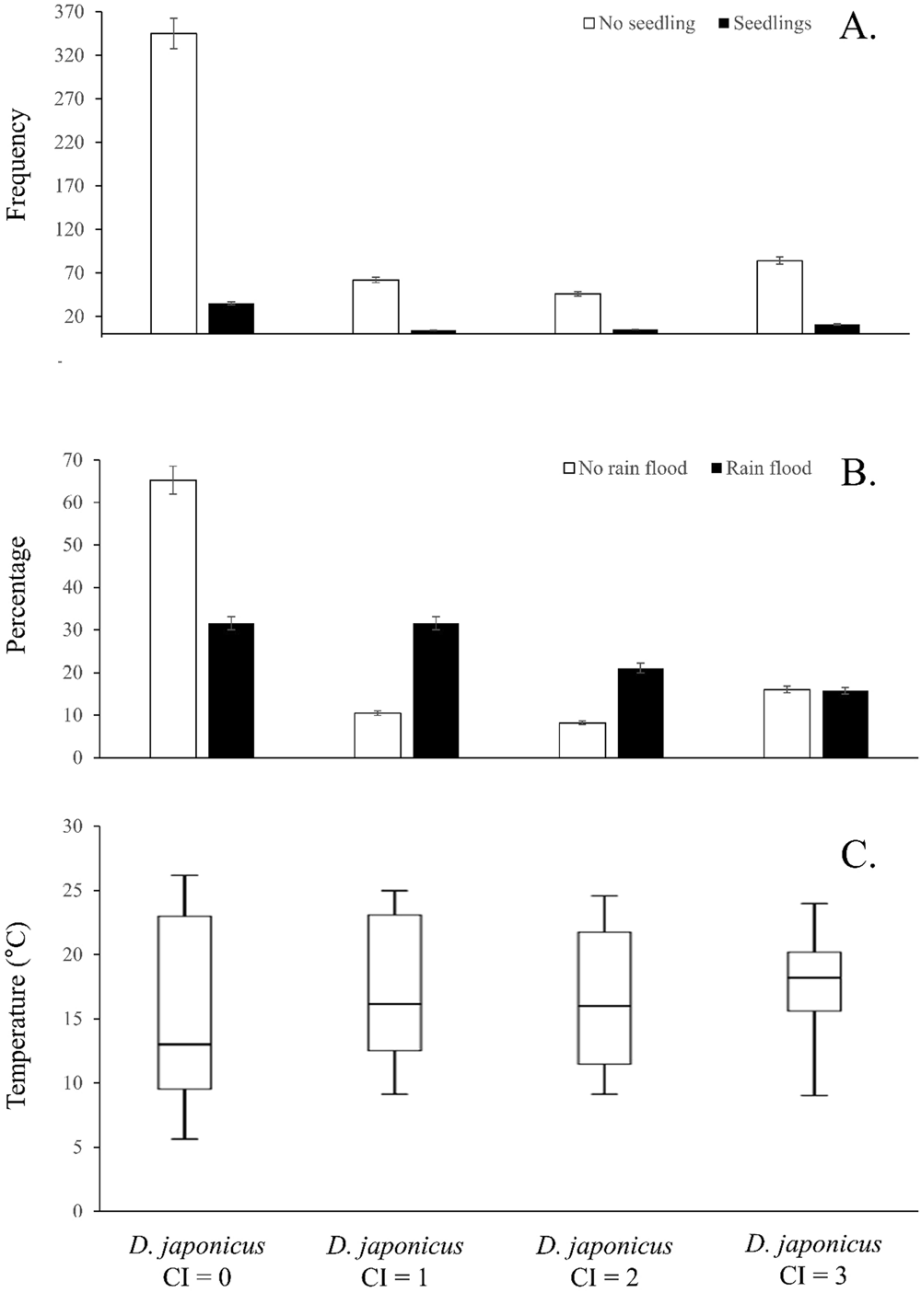
When summing the R^2^ values from a Pearson’s Correlation for each of the variables with the three other variables representative of the presence and activity of *Dryophytes japonicus*, the highest score goes to the CI variable. It is thus used for the graphical representation of the data, here presented for temperature, pre-seedling and rain flood. (**A**) *D. japonicus* calling in relation to rice pre-seedlings, (**B**) *D. japonicus* calling index in relation to rain flood, (**C**) *D. japonicus* calling index in relation to temperature.

The effects of agricultural and environmental factors on the presence of either species at the survey points and rice-paddy complexes were statistically significant for occurrence of *D. japonicus* at survey points: χ^2^_(7)_ = 184.66, *p* < 0.001; occurrence of *D. suweonensis* at survey points: χ^2^_(7)_ = 98.87, *p* < 0.001; occurrence of *D. japonicus* at complexes: χ^2^_(7)_ = 74.36, *p* < 0.001; and occurrence of *D. suweonensis* at complexes: χ^2^_(7)_ = 217.04, *p* < 0.001. The models explained between 16.7 and 42.4% (Nagelkerke R^2^) of the variance in occurrence and correctly classified between 73.0 and 87.5% of cases. For both species, and at both spatial scales, the cultivation phase was significant (Fig. 2). Temperature and rain flood were also significant for *D. japonicus* at both site and complex scale (Fig. 3), while the only additional significant variable for *D. suweonensis* was temperature at the rice-paddy complexes, and rice paddy-complex at the single survey point scale (Table 1).

**Table 1:** Results of Binominal regressions conducted to assess the significance of environmental and agricultural variables on occurrence of *Dryophytes suweonensis* and *D. japonicus* at single survey points and within rice-paddy complexes (*n* = 592).

	B	S.E.	Wald	df	*p*-value
**Occurrence** ***D. japonicus*** **at single survey points**					
Temperature	−0.06	0.03	5.54	1	**0.019**
Humidity	−0.02	0.01	3.73	1	0.053
Cultivation phase	1.51	0.15	106.67	1	**<0.001**
Pre-seedlings	−0.16	0.35	0.12	1	0.664
Complex x survey point	0.01	0.01	2.31	1	0.069
Complex	−0.22	0.15	2.27	1	0.132
Rain flood	2.42	0.54	19.69	1	**<0.001**
**Occurrence** ***D. suweonensis*** **at single survey points**					
Temperature	−0.04	0.04	1.08	1	0.298
Humidity	−0.01	0.01	0.06	1	0.938
Cultivation phase	1.57	0.24	43.03	1	**<0.001**
Pre-seedlings	0.81	0.48	2.77	1	0.096
Complex x survey point	−0.01	0.01	3.45	1	0.063
Complex	0.47	0.22	4.46	1	**0.035**
Rain flood	−17.80	9089.36	0.01	1	0.998
**Occurrence** ***D. japonicus*** **in complex**					
Temperature	0.06	0.02	6.71	1	**0.010**
Humidity	−0.01	0.01	1.22	1	0.255
Cultivation phase	0.43	0.11	15.87	1	**<0.001**
Pre-seedlings	−0.11	0.32	0.12	1	0.729
Complex x survey point	−0.01	0.01	0.06	1	0.804
Complex	−0.10	0.14	0.55	1	0.458
Rain flood	2.47	1.03	5.71	1	**0.017**
**Occurrence** ***D. suweonensis*** **in complex**					
Temperature	0.13	0.03	22.53	1	**<0.001**
Humidity	−0.01	0.01	0.31	1	0.578
Cultivation phase	0.82	0.13	37.45	1	**<0.001**
Pre-seedlings	0.54	0.36	2.26	1	0.133
Complex x survey point	−0.01	0.01	0.36	1	0.546
Complex	0.29	0.16	3.36	1	0.067
Rain flood	0.33	0.61	0.29	1	0.586

These results demonstrate that for both species, and despite the different scales used, the occurrence at survey points was the variable least affected by agricultural practices, followed by the occurrence at rice-paddy complexes, and finally by the number of individuals and the CI. When considering *D. japonicus* alone, the variation was minimal for occurrence at survey points, with three factors of significance for the number of calling individuals and CI (*n* = 5). When *D. suweonensis* was considered alone in the analysis, a single environmental variable was significant for occurrence at survey points, and the maximum number of factors for occurrence in rice-paddy complex was three.

#### Generalised Linear Model

The model assessing the effect of variables on the number of calling *D. suweonensis* was a good fit for the data (Omnibus Test; Likelihood ratio *χ*^2^_(44)_ = 197.46, *p* < 0.001). The results of the test (Table 2) show that temperature and cultivation phase are significant (Fig. 2). Furthermore, both site nested within complex and rice-paddy complex are significant for the number of calling *D. suweonensis*, but here only highlight the variation in population size between sites.

**Table 2:** Results of the Generalised Linear Model conducted to assess the significance of environmental and agricultural variables on the number of calling *Dryophytes suweonensis* at sites (*n* = 592).

	χ²	df	*p*-value
Site(Complex)	52.92	30	**0.006**
Complex	43.54	9	**<0.001**
Pre-seedlings	1.62	1	0.204
Rain flood	1.27	1	0.259
Temperature	4.29	1	0.038
Humidity	1.08	1	0.299
Cultivation phase	67.47	1	**<0.001**

#### GLM

The model to test whether variables fit into the model showed that all but one variables were valid and significant, with humidity the least informative: cultivation phase: Λ = 0.77, *F*_(16,1653)_ = 9.34, *p* < 0.001; pre-seedling Λ = 0.98, *F*_(4,541)_ = 2.46, *p* = 0.044; rain flood Λ = 0.97, *F*_(4,541)_ = 3.72, *p* = 0.005; temperature Λ = 0.98, *F*_(4,541)_ = 2.91, *p* = 0.021; humidity Λ = 0.99, *F*_(4,541)_ = 1.97, *p* = 0.098; complex(survey point) Λ = 0.59, *F*_(156,2158)_ = 2.46, *p* = 0.044 and complex Λ = 0.72, *F*_(4,541)_ = 2.84, *p* = 0.048. The results of the GLM (Table 3) show that temperature, cultivation phase, pre-seedlings, site nested within complex and rice-paddy complex are significant (Fig. 3). Survey points nested within complex are also significant for both species, but here only highlight the variation in population size between sites.

**Table 3:** Results of the General Linear Model conducted to assess the significance of environmental and agricultural variables on the CI of *D. japonicus* at sites (*n* = 592).

	*df*	*χ²*	*F*	*p*-value
Temperature	1	7.34	7.92	**0.005**
Humidity	1	0.01	0.01	0.915
Cultivation phase	4	25.71	27.74	**<0.001**
Pre-seedlings	1	4.02	4.34	**0.038**
Complex(Site)	39	2.23	2.41	**<0.001**
Complex	9	1.98	1.46	0.476
Rain flood	1	9.3	10.03	**0.002**
Error	544	0.93		

Based on both regressions and Linear Models it is clear that the critical factor for the occurrences of the two species is the cultivation phase, although *D. suweonensis* reaches peak calling activity later than *D. japonicus* (Fig. 2). The fallow and ploughing phases were characterised by a very low calling index for *D. japonicus*, and a single calling male *D. suweonensis*. The tilling phase saw a large increase in calling activity for *D. japonicus*, up to CI = 3, but *D. suweonensis* was still calling at only 2.6% of survey points.

*Dryophytes japonicus* calling is also significantly influenced by other factors: temperature, pre-seedlings and rain flood (Fig. 3). Once CI = 0 is excluded, because of its match with the pre-lekking period, the CI increases with the presence of pre-seedlings (*n* = 55; CI = 1 reaches 7.3, CI = 2 reaches 9.1% and CI = 3 reaches 20.0%). For rain flood, the relation with *D. japonicus* calling activity is less intuitive as the number of paddies flooded by rain (*n* = 19) decreases once paddies are flooded by agricultural water, but the effect is clear as CI = 0 represents 31.6% of cases, and the three other CI combined represent 68.4% of cases.

### Seasonal variations in calling activity

**Figure 4:**
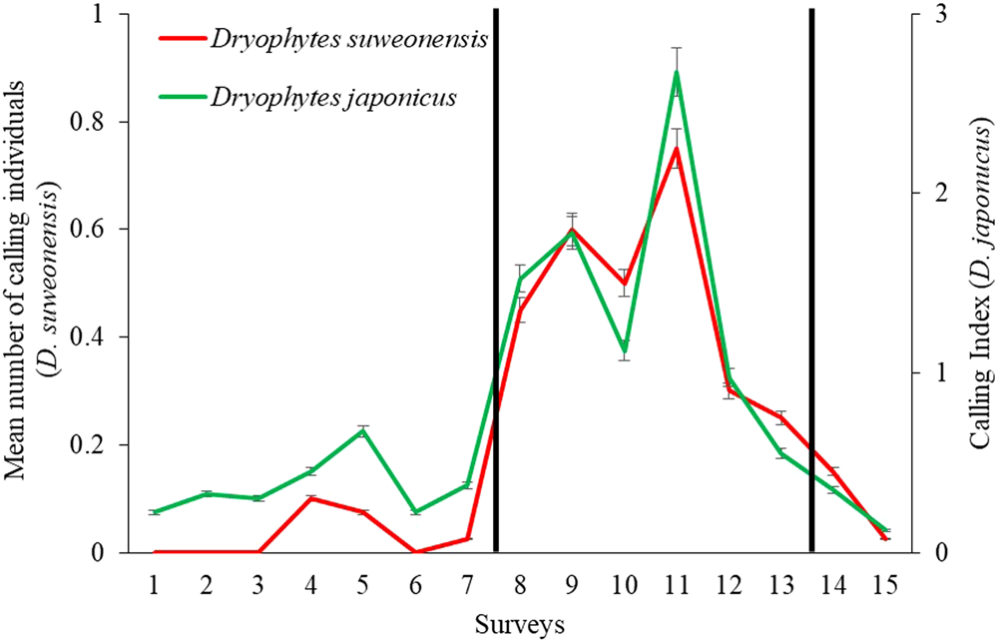
Mean number of calling individuals and the mean CI over the study period, covering 60 days, and starting on 16 April 2014. The lekking activity is divided in pre-lekking for surveys 1 to 7, lekking for surveys 8 to 13 and post lekking for surveys 14 and 15. The vertical black bars represent separations between the three lekking phases.

Following the definition of the lekking phases, we found that during the pre-lekking period, (*n* = 280; from 40 survey points surveyed eight times), there were 0.03 ± 0.24 (mean ± SD) calling male *D. suweonensis* and a median CI of zero for *D. japonicus*. For the lekking period (*n* = 200), an average of 0.52 ± 1.11 *D. suweonensis* were calling and the median CI was two for *D. japonicus*. Finally, for the post-lekking period, *n* = 120, a total of 0.14 ± 0.51 individuals *D. suweonensis* were calling, and the median CI for *D. japonicus* was zero again. The median value of CI was zero for all time periods for *D. suweonensis*, due to very low numbers of individuals (Fig. 4). During agricultural flooding, the percentage of survey points reaching CI = 3 for *D. japonicus* was at 65.5%, matching the transition between the pre-lekking and lekking periods for the species, although *D. suweonensis* was calling at 32.7% of survey points only. The calling activities of both species peaked with rice plantation stage (Fig. 2).

### Pre- and post-lek

The results of the binary logistic regressions for pre-lekking (Table 4) showed that the calling activity of *D. japonicus* was significantly influenced by temperature, while the calling activity of *D. suweonensis* was significantly related to temperature and season. The binary logistic regressions for post-lekking (Table 5) displayed the opposite trend, with *D. japonicus* call production significantly related to agricultural flooding only, while *D. suweonensis* was significantly influenced by season, humidity, and agricultural flooding. For the pre-lekking period, temperature was significant for both species, while flooding was the only significant variable for both species during the post-lekking period. These two variables are limiting factors at these two time periods.

**Table 4:** Binary logistic regression explaining the breeding effort in relation to abiotic variables during the pre-lekking period (surveys 1 to 7).

	*Dryophytes japonicus*					*Dryophytes suweonensis*				
	*B*	S.E.	Wald	*df*	*P-value*	*B*	S.E.	Wald	*df*	*P-value*
Season	0.04	0.02	2.64	1	0.104	0.27	0.12	4.6	1	**0.032**
Temperature	0.33	0.06	25.63	1	**<0.001**	0.63	0.22	8.7	1	**0.003**
Humidity	0.02	0.01	2.05	1	0.152	−0.01	0.03	0.01	1	0.971
Cultivation phase	0.23	0.17	1.89	1	**0.169**	−0.11	0.38	0.08	1	0.767

Each species was set as dependent variables and season, temperature, humidity and cultivation phases were set as independent variables (*n* = 280 for both species).

**Table 5:** Binary logistic regression explaining the breeding effort in relation to abiotic variables during the post-lekking period (survey 14–15).

	*Dryophytes japonicus*					*Dryophytes suweonensis*				
	*B*	S.E.	Wald	*df*	*P-value*	*B*	S.E.	Wald	*df*	*P-value*
Season	−0.04	0.02	2.78	1	0.095	−0.05	0.02	5.04	1	**0.025**
Temperature	−0.13	0.26	0.26	1	0.610	0.05	0.26	0.03	1	0.854
Humidity	−0.05	0.05	1.14	1	0.286	−0.12	0.05	5.64	1	**0.018**
Agricultural flooding	−1.55	0.43	13.13	1	**<0.001**	−0.97	0.41	5.57	1	**0.018**

Each species was set as dependent variables and season, temperature, humidity and agricultural flooding were set as independent variables (*n* = 120 for both species).

The descriptive statistics for significantly influential variables during the pre-lekking period (Table 6) indicated a higher temperature for *D. japonicus. Dryophytes suweonensis* calling was associated with a higher temperature and the season. For the post-lekking period, the calling activity of both treefrog species was influenced by a higher ratio of flooded rice paddies. The latter was also related to a lower humidity, and the calling activity also diminished in relation to the season.

**Table 6:** Descriptive statistics for pre- and post-lekking for surveys 1 to 7 (*n* = 280) for *Dryophytes japonicus* and *D. suweonensis*, respectively, and surveys 14 and 15 (*n* = 120). “+” stands for “presence and “−” for absence of the species.

		Mean	St. dev.	Min.	Max.	Range
**Pre-lekking**	***D. japonicus***					
Temperature (°C)	−	10.25	7.35	5.60	16.80	11.20
	+	10.60	6.03	7.50	16.70	9.20
	***D. suweonensis***					
Temperature (°C)	−	10.67	2.44	5.60	16.70	11.10
	+	13.56	2.54	10.00	16.80	6.80
Season (days)	−	10.24	6.76			
	+	13.50	4.22			
**Post-lekking**	***D. japonicus***					
Agricultural flooding (%)	−	29%	46%			
	+	61%	49%			
	***D. suweonensis***					
Agricultural flooding (%)	−	41%	50%			
	+	57%	50%			
Season (days)	−	57.38	10.15	40.00	64.00	24.00
	+	53.00	11.38	40.00	64.00	24.00
Humidity (%)	−	89.76	4.37	78.60	99.90	21.30
	+	87.75	4.14	77.10	97.60	20.50

### Lekking period

The results of the multinomial logistic regression demonstrated a difference between the two species in the factors important during the full lek period. For *D. japonicus*, temperature and cultivation phase were significant, while no variable was significant for *D. suweonensis* (Table 7). The highest temperature corresponded to CI = 1 for *D. japonicus* and to two calling individuals for *D. suweonensis* (18.89 ± 3.76 °C, or CI = 1, Table 8). High values for agricultural flooding and tilling were associated with the highest calling index for *D. japonicus*, but the trend was less distinct for rain flooding because of the short seasonality of the variable, and for which the highest values were correlated with CI = 1.

**Table 7:** Multinomial logistic regression explaining the breeding effort in relation to abiotic variables during the lekking period (surveys 8 to 13; *n* = 200).

	*Dryophytes japonicus*				*Dryophytes suweonensis*			
	AIC	Chi-Square	df	*P-value*	AIC	Chi-Square	df	*P-value*
Season	308.96	1.09	3	0.778	32.65	0.93	3	0.336
Temperature	327.84	19.97	3	**<0.001**	32.87	1.14	3	0.285
Humidity	311.48	3.62	3	0.688	32.07	0.35	3	0.555
Cultivation phase	404.16	92.69	15	**<0.001**	38.18	6.45	5	0.265

Each species was set as dependent variables and season, temperature, humidity and cultivation phase were set as independent variables.

**Table 8:** Descriptive statistics for statistically significant variables for *Dryophytes japonicus* and *D. suweonensis* for the lekking period (surveys 8 to 13; *n* = 200).

	CI	Mean	Median	Std. Dev.	Min.	Max.
***Dryophytes japonicus***						
Temperature	0	17.44	15.80	4.25	12.20	24.00
	1	19.05	18.90	4.48	12.50	24.50
	2	19.01	19.90	3.69	14.00	23.60
	3	18.47	18.50	2.75	12.60	24.00
Tilling	0	0.78	1.00	0.42		
	1	0.83	1.00	0.39		
	2	0.00	0.00	0.00		
	3	0.98	1.00	0.12		
Rain flooding	0	0.07	0.00	0.25		
	1	0.09	0.00	0.29		
	2	0.00	0.00	0.00		
	3	0.02	0.00	0.12		
Agricultural flooding	0	0.64	1.00	0.48		
	1	0.87	1.00	0.34		
	2	1.00	1.00	0.00		
	3	1.00	1.00	0.00		
***Dryophytes suweonensis***						
Temperature	0	18.64	18.55	3.89	12.20	24.20
	1	18.16	17.30	3.39	13.10	24.50
	2	17.58	18.50	2.91	12.60	21.20

No CI = 3 were collected for *D. suweonensis*. Cultivation phases are divided into the different categories for ease of understanding. Despite not reaching significance, temperatures are presented for *D. suweonensis* as an indicator.

**Figure 5:**
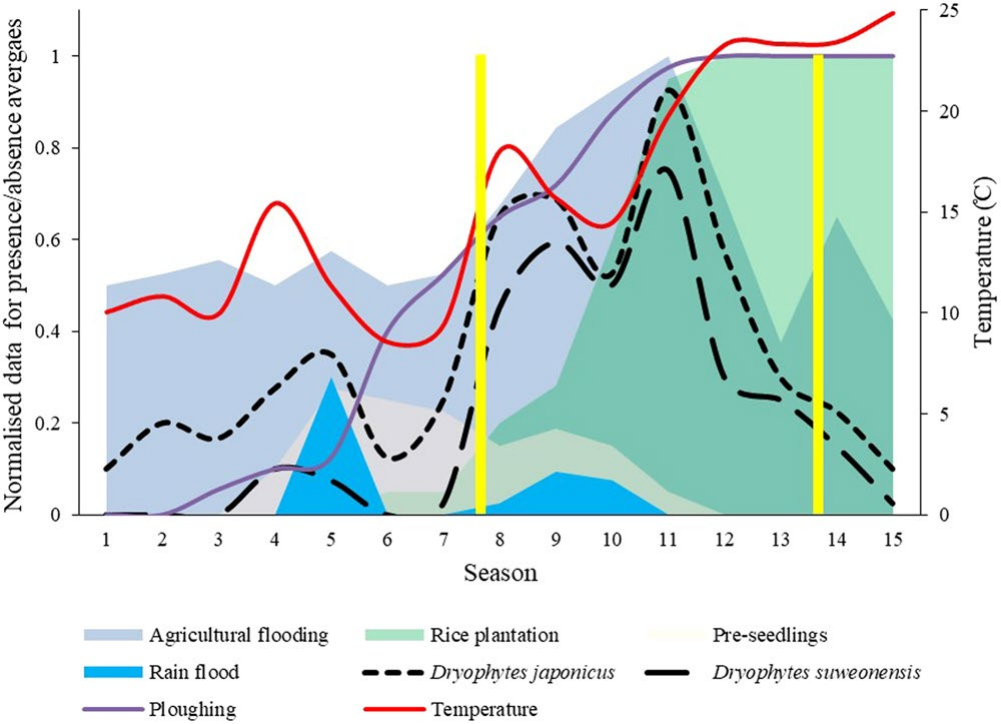
Calling activity of both Hylid species in function of all significant agricultural and environmental variables. The first peak in presence for the two Hylid species is due to an overlap between rain floods and pre-seedling, the second peak is correlated with a temperature increase and the last and highest peak is correlated with the overlap of agricultural flooding and rice plantation. The yellow vertical bars denote the lekking period.

Graphical presentation of variables influential to the calling activity of the two species (Fig. 5) shows the close association between calling, and rain flooding and temperature for the pre-lekking period, confirmed through Pearson Correlations (*n* = 592, *R²* = 0.29, *p* < 0.001).

Finally, the Pearson correlation tests for the influence of the weekly calling activity of the two species on each other were not found to be significant (*r* < 0.32; *p*-value > 0.067), with the exception of week 13 (*r* = 0.80, *p-*value < 0.001). Replicate 13 is the last of the lekking period, and we speculate that the highest number of calling individuals of both species on that week was related to the optimal breeding conditions, rather than the influence of the two species on each other.

## Discussion

Our results highlight similarities and divergences in agro-environmental preferences between the numerous *Dryophytes japonicus* and the endangered *D. suweonensis*. According to our hypothesis, *D. japonicus* begins breeding, here assessed through calling behaviour^71–73^, before rice plantation and therefore earlier than *D. suweonensis*. This matches with the expected behaviour of a species hydroperiodically restrained during its evolution, and thus inclined towards the opportunistic use of water bodies. In contrast, *D. suweonensis* generally does not start breeding before rice is planted, which corresponds to the expected behaviour of a species that would have been relying on seasonal floods for breeding, over evolutionary times. Both species reach peak calling activity once rice seedlings have been planted and this matches the optimal hydroperiod. However, as *D. japonicus* starts breeding as soon as fields are ploughed, or even earlier if there is rainwater partially flooding rice paddies, egg masses may be negatively impacted by subsequent ploughing, tilling and planting.

Although the breeding phenology of both species is linked to agricultural practices, their effects on the two species may appear contradictory with the current population dynamics, i.e. positive for *D. suweonensis* and negative for *D. japonicus*. As a general rule during the entire breeding season, and apart from the agricultural phases, *D. japonicus* was more sensitive to environmental variations than *D. suweonensis*, this may be a potential artefact of the prolonged breeding period, and highlighting a difference in behavioural plasticity between the two species. Generally, the variables important for the breeding activity of *D. japonicus* were temperature and rain flood. Another commonality between the two species is that breeding activity is not linked to humidity, the opposite of commonly held expectations.

The splitting of the breeding period into pre-lekking, lekking, and post-lekking is consistent with the lekking activity described for a number of species^66^, although the presence of female *D. suweonensis* for the only purpose of fertilisation would have to be determined to confirm a match with the definition of lekking species^67,74,75^. When limited to the pre-lekking period, temperature is important to both species, while season is also important to *D. suweonensis*. The increase in calling activity during the lekking period is remarkable for its overlap with rice plantation, while the decrease in breeding activity is closely linked to the decrease in agricultural flooding after the peak calling activity. The calling activity of *D. japonicus* during the post-lekking period is not related to any variables, and is hypothesised to decrease following a drop in hormonal level^76–78^, although it may also be related to the more vigorous behaviour of *D. japonicus* in comparison to *D. suweonensis*^79^. Both season and humidity are important for *D. suweonensis* during the post-lekking period, highlighting the end of the breeding season for the species. The shorter breeding season for *D. suweonensis* may partially explain the difference in population sizes between the two species, as *D. japonicus* has more time to deposit eggs and females can lay eggs more than once^68^.

In this study, *D. suweonensis* started breeding later than *D. japonicus*, and *D. suweonensis* may require the availability of large pools of water to start producing advertisement calls^52^. Alternatively, the calling activity of *D. suweonensis* may be triggered by some levels of temperature or photoperiod^70,80^, which coincides with the season when farmers start planting rice. From our results, and if such a trigger variable does exist, we can estimate a trigger temperature *circa* 12.87 ± 0.27 °C for *D. suweonensis*. The fact that *D. suweonensis* is now entirely restricted to rice paddies for breeding^50^, and that rice farming may not start as early as natural wetland flooding, this restraint on its breeding potential, is another possible explanatory factor for its endangered status.

*Dryophytes suweonensis* may benefit from farming activities, which could at the same time negatively impact the first eggs and larvae of *D. japonicus* deposited before agricultural flooding. If these larvae fail to develop, newly hatched *D. suweonensis* tadpoles will not be subject to competition with older, and more developed *D. japonicus* tadpoles. This is important both for indirect competition, as tadpoles of both species extract the same resources, and for interference competition as *Dryophytes* tadpoles are cannibalistic.

The fact that our results consistently point to a higher number of significant factors for *D. japonicus* than for *D. suweonensis* is possibly due to the length of the breeding season. Species with longer breeding periods are generally more dependent on their environment than species with a shorter breeding season, *i.e*. explosive breeders^81,82^. Because of a shorter seasonal window at breeding sites by male *D. suweonensis*, variation in calling activity in relation to the agro-environment may be less detectable than for *D. japonicus*. Also, because of the greater numbers of male *D. japonicus*, more variations can be observed and would highlight the need for a larger sample size for *D. suweonensis* to present increased breeding variability.

The rapid decrease in the number of calling individuals in relation to the agricultural flooding of rice paddies warrants additional research. Although possibly coincidental, it seems that both *Dryophytes* species would benefit from a longer hydroperiod for tadpole development. In regions with rich water resources this would also be beneficial to rice crops as flooding prevents weed growth in rice paddies. Pesticide use has been associated with the decline of *D. suweonensis*^83^ and longer flood times would reduce the need for its usage. The conservation of *D. suweonensis* would appear to require an earlier flooding of rice paddies, and we therefore recommend flooding during the fallow phase^7^. This procedure would also benefit the conservation of a number of other species, including the endangered Seoul Golden Frog (*Pelophylax chosenicus*), commonly sympatric with *D. suweonensis*.

Rice paddies are not as biologically diverse as natural wetlands, and neither are they as economically productive as industries or cities. As a result, agricultural wetlands are easily sold for development, and encroachment is frequent, facilitated by the close proximity to cities, increasing further the risks to amphibian’s survival^84,85^. The low economical value of rice paddies in developed countries is consequently an indirect reason for the increasing scarcity of amphibian secondary habitats. However, rice paddies are the only breeding ground for some species, and their conservation has to be a priority. Finally, an emphasis on the protection of rice paddies as a breeding habitat for species is required. We urge the development of farming guidelines for sites where endangered species occur, under international conservation frameworks compatible with agriculture, such as RAMSAR.

## Electronic supplementary material

Supplementary information
