# Supplementary material for: Relationship between agro-environmental variables and breeding Hylids in rice paddies
Source: Sci Rep. 2018 May 23;8:8049. doi: 10.1038/s41598-018-26222-w (PMC5966434; doi:10.1038/s41598-018-26222-w)
Supplement: Supplementary file 1 — Supplementary information [file 41598_2018_26222_MOESM1_ESM.pdf]

Relationship between agro-environmental variables and breeding Hylids in rice paddies

Amaël Borzée[1,3], Kyongman Heo[2,3] and Yikweon Jang[3, 4,*]

[1] Laboratory of Behavioral Ecology and Evolution, School of Biological Sciences, Seoul National University, 08826, Republic of Korea

[2] College of Natural Science, Sangmyung University, Seoul 03016, Korea

[3] Department of Life Sciences and Division of EcoScience, Ewha Woman's University, Seoul, 03760, Republic of Korea

[4] Interdisciplinary Program of EcoCreative, Ewha Womans University, Seoul, 03760, Republic of Korea

* Correspondence to jangy@ewha.ac.kr

Supplementary materials 1*:*

P-values for pairwise comparisons of main effects of the repeated ANOVA on the bi-weekly surveys at each of the sites. The framed box represents the replicates which fall in a different category compared with the others.

Replicates	1	2	3	4	5	6	7	8	9	10	11	12	13	14	15
1				0.103	0.323		0.323	0.095	< 0.001	0.001	< 0.001	0.003	0.023	0.083	0.323
2				0.103	0.323		0.323	0.095	< 0.001	0.001	< 0.001	0.003	0.023	0.083	0.323
3				0.103	0.323		0.323	0.095	< 0.001	0.001	< 0.001	0.003	0.023	0.083	0.323
4	0.103	0.103	0.103		0.743	0.103	0.083	0.209	0.004	0.016	0.001	0.103	0.244	0.643	0.262
5	0.323	0.323	0.323	0.743		0.323	0.534	0.183	0.005	0.013	0.001	0.083	0.197	0.520	0.534
6				0.103	0.323		0.323	0.095	< 0.001	0.001	< 0.001	0.003	0.023	0.083	0.323
7	0.323	0.323	0.323	0.083	0.534	0.323		0.117	< 0.001	0.002	< 0.001	0.010	0.048	0.168	0.999
8	0.095	0.095	0.095	0.209	0.183	0.095	0.117		0.577	0.853	0.220	0.509	0.384	0.271	0.111
9	< 0.001	< 0.001	< 0.001	0.004	0.005	< 0.001	< 0.001	0.577		0.440	0.337	0.017	0.014	0.005	< 0.001
10	0.001	0.001	0.001	0.016	0.013	0.001	0.002	0.853	0.440		0.142	0.088	0.115	0.037	0.001
11	< 0.001	< 0.001	< 0.001	0.001	0.001	< 0.001	< 0.001	0.220	0.337	0.142		0.002	0.008	0.003	< 0.001
12	0.003	0.003	0.003	0.103	0.083	0.003	0.010	0.509	0.017	0.088	0.002		0.623	0.183	0.003
13	0.023	0.023	0.023	0.244	0.197	0.023	0.048	0.384	0.014	0.115	0.008	0.623		0.160	0.027
14	0.083	0.083	0.083	0.643	0.520	0.083	0.168	0.271	0.005	0.037	0.003	0.183	0.160		0.133
15	0.323	0.323	0.323	0.262	0.534	0.323	0.999	0.111	< 0.001	0.001	< 0.001	0.003	0.027	0.133	
